# Untangling Species-Level Composition of Complex Bacterial Communities through a Novel Metagenomic Approach

**DOI:** 10.1128/mSystems.00404-20

**Published:** 2020-07-28

**Authors:** Christian Milani, Giulia Alessandri, Marta Mangifesta, Leonardo Mancabelli, Gabriele Andrea Lugli, Federico Fontana, Giulia Longhi, Rosaria Anzalone, Alice Viappiani, Sabrina Duranti, Francesca Turroni, Renato Costi, Alfredo Annicchiarico, Andrea Morini, Leopoldo Sarli, Maria Cristina Ossiprandi, Douwe van Sinderen, Marco Ventura

**Affiliations:** a Laboratory of Probiogenomics, Department of Chemistry, Life Sciences, and Environmental Sustainability, University of Parma, Parma, Italy; b Microbiome Research Hub, University of Parma, Parma, Italy; c Department of Veterinary Medical Science, University of Parma, Parma, Italy; d GenProbio srl, Parma, Italy; e Department of Medicine and Surgery, University of Parma, Parma, Italy; f APC Microbiome Institute and School of Microbiology, Bioscience Institute, National University of Ireland, Cork, Ireland; Northern Arizona University

**Keywords:** metagenomics, ITS, profiling

## Abstract

16S small-subunit (SSU) rRNA gene-based bacterial profiling is the gold standard for cost-effective taxonomic reconstruction of complex bacterial populations down to the genus level. However, it has been proven ineffective in clinical and research settings requiring higher taxonomic resolution. We therefore developed a bacterial profiling method based on the internal transcribed spacer (ITS) region employing optimized primers and a comprehensive ITS database for accurate cataloguing of bacterial communities at (sub)species resolution. Performance of the microbial ITS profiling pipeline was tested through analysis of host-associated, food, and environmental matrices, while its efficacy in clinical settings was assessed through analysis of mucosal biopsy specimens of colorectal cancer, leading to the identification of putative novel biomarkers. The data collected indicate that the proposed pipeline represents a major step forward in cost-effective identification and screening of microbial biomarkers at (sub)species level, with relevant impact in research, industrial, and clinical settings.

**IMPORTANCE** We developed a novel method for accurate cataloguing of bacterial communities at (sub)species level involving amplification of the internal transcribed spacer (ITS) region through optimized primers, followed by next-generation sequencing and taxonomic classification of amplicons by means of a comprehensive database of bacterial ITS sequences. Host-associated, food, and environmental matrices were employed to test the performance of the microbial ITS profiling pipeline. Moreover, mucosal biopsy samples from colorectal cancer patients were analyzed to demonstrate the scientific relevance of this profiling approach in a clinical setting through identification of putative novel biomarkers. The results indicate that the ITS-based profiling pipeline proposed here represents a key metagenomic tool with major relevance for research, industrial, and clinical settings.

## Abstract (precis)

We developed a novel method for accurate cataloguing of bacterial communities at (sub)species level involving amplification of the internal transcribed spacer (ITS) region through optimized primers, followed by next-generation sequencing and taxonomic classification of amplicons by means of a comprehensive database of bacterial ITS sequences. Host-associated, food, and environmental matrices were employed to test the performance of the microbial ITS profiling pipeline. Moreover, mucosal biopsy samples from colorectal cancer patients were analyzed to demonstrate the scientific relevance of this profiling approach in a clinical setting through identification of putative novel biomarkers. The results indicate that the ITS-based profiling pipeline proposed here represents a key metagenomic tool with major relevance for research, industrial, and clinical settings.

## INTRODUCTION

The relevance of microbial communities, i.e., the microbiota, has been demonstrated for a range of environmental and host-associated ecological niches, including soil, water, and air, as well as in animal and human body sites (1–6). Due to their impact on many physiological and immunological aspects of their hosts, microbial populations harbored by humans have been associated with various diseases and disorders. Limitations in the taxonomic resolution of the commonly used microbial profiling approach based on the 16S rRNA gene have hampered identification of microbial biomarkers and cost-effective, population-wide screening for bacterial species of clinical relevance.

Currently, shotgun and long-read sequencing methods provide higher taxonomic resolution, although their higher cost represents an important limitation compared to the use of 16S rRNA-based microbial profiling approach (7–9). Moreover, the absence of a PCR amplification step may prevent retrieval of sufficient microbial DNA for analysis of host-associated, low-abundance bacterial communities due to interfering levels of eukaryotic DNA (7).

To overcome the limitations of 16S rRNA gene microbial profiling and shotgun metagenomics, we present here a pipeline for reliable and cost-effective profiling of microbial communities at the (sub)species level. In detail, we extended the internal transcribed spacer (ITS)-profiling approach described previously for bifidobacteria and lactobacilli (10–12) to all members of the domain *Bacteria*. ITS sequences are characterized by higher variability than 16S rRNA genes, thus allowing (sub)species taxonomic resolution when employed for metagenomic profiling purposes (10–12). Remarkably, the ITS profiling method combines the lower cost and high sensitivity of a marker gene amplification approach with the resolution of shotgun metagenomics (7, 8).

## RESULTS AND DISCUSSION

### The bacterial ITS profiling pipeline.

Our ITS profiling pipeline encompasses universal ITS primers for bacteria, a database including ITS sequences retrieved from bacterial genomes currently available in the NCBI database, and a ready-to-use bioinformatic script for QIIME v.2.

Genomic sequences of 120,748 bacterial strains were retrieved from NCBI and processed for prediction of rRNA loci through RNAmmer (13) in order to build a database encompassing all retrieved 16S-ITS-23S genomic regions. The resulting universal microbial ITS database (UMID) comprises 131,795 sequences covering 10,361 bacterial species. UMID is available in both FASTA and QIIME v.2 artifact formats (http://probiogenomics.unipr.it/pbi/) and was used for all analyses performed in this study. Moreover, an alternative database named UMID-RefSeq is also provided in QIIME v.2 artifact format as part of the script package (http://probiogenomics.unipr.it/pbi/). The latter was obtained by extracting the 16S-ITS-23S region from genomes available in the NCBI RefSeq database. Notably, the average nucleotide identity (ANI) matrix precomputed and updated by NCBI is used for correction of taxonomic assignments, leading to 91,309 database entries which represent 4,202 bacterial species. A list of bacterial species covered by UMID and UMID-RefSeq is available in Data Set S1. Furthermore, a script to generate an updated UMID-RefSeq is available for download (http://probiogenomics.unipr.it/pbi/).

10.1128/mSystems.00404-20.5 DATA SET S1 UNI_ITS_fw/UNI_ITS_rv primers and UMID statistics. Download Data Set S1, XLSX file, 0.6 MB. Copyright © 2020 Milani et al. 2020 Milani et al. This content is distributed under the terms of the Creative Commons Attribution 4.0 International license.

**FIG 1:**
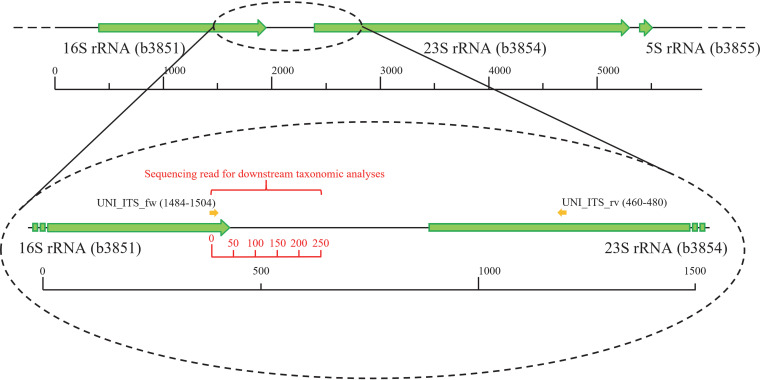
Positions of primers UNI_ITS_fw and UNI_ITS_rv within the ribosomal locus of Escherichia coli K-12 strain MG1655.

Universal primers for bacteria were designed through alignment of 16S-ITS-23S sequences in the UMID and processing through PrimerProspector (14) and the SILVA database v.132 (15) Test Probe web application (https://www.arb-silva.de/search/testprobe/). Primers were designed at the 3′ end of the 16S rRNA sequence (UNI_ITS_fw, 5′-KRGGRYKAAGTCGTAACAAG-3′) and the 5′ end of the 23S rRNA sequence (UNI_ITS_rv, 5′-TTTTCRYCTTTCCCTCACGG-3′), corresponding to positions 1484 to 1504 and 460 to 480 of 16S and 23S rRNA genes of Escherichia coli K-12 strain MG1655, respectively, matching conserved regions of ribosomal genes in order to maximize taxonomic coverage (Fig. 1). In this regard, 100 16S rRNA and 100 23S rRNA gene sequences spanning a wide range of bacterial taxonomy were retrieved from the SILVA database and aligned using ClustalX (16) in order to identify a suitable conserved region at the 3′ end of the desired amplicon. Alignments are available in Clustal format as Data Sets S5 and S6 for 16S rRNA and 23S rRNA sequences, respectively. Primer sequences can be found at position 1680 in the 16S rRNA sequence alignment and at position 718 in the 23S rRNA sequence alignment. Furthermore, PrimerProspector (14) and the SILVA database v.132 (15) Test Probe web application (https://www.arb-silva.de/search/testprobe/) were used to identify mismatches in alignments with specific taxonomic ranks (on a phylum and class basis), leading to the iterative introduction of IUPAC bases in the primer sequence in order to maximize taxonomic coverage.

*In silico* PCR amplification of the UMID with UNI_ITS_fw/UNI_ITS_rv primers by means of FastPCR software (17) revealed that the average amplicon size is 972.6 ± 153.7 nucleotides (nt). The sequencing technology used (Illumina, San Diego, CA, USA) has a recommended fragment or amplicon size of 200 to 500 bp, with larger fragment/amplicon sizes, such as 1,000 bp and higher, causing increasingly lower sequencing efficiency and lower sequence yield (18). For this reason, we amended the standard library preparation protocol by employing 15 μl of Agencourt AMPure XP DNA purification beads (Beckman Coulter Genomics GmbH, Bernried, Germany) for the first purification step in order to remove primer dimers. In addition, we performed the second purification step using 30 μl of the above-mentioned purification beads. With these adaptations, we did not observe any sequencing efficiency issues with libraries generated from samples sequenced as part of the current work, especially with the R1/forward reads, confirming recently published data (19). The designed primers target the 16S rRNA-proximal side of the amplicons for taxonomic assignment (Fig. 1). Thus, in the case of paired-end sequencing of the PCR amplicon, only the read starting with the UNI_ITS_fw primer sequence is used for taxonomic assignment in bioinformatic analyses. Alignment of primers against the bacterial sequences contained in the SILVA REF small-subunit (SSU) and large-subunit (LSU) databases, v.132, with five mismatches allowed through the testProbe web application (https://www.arb-silva.de/search/testprobe/) is reported in the bacterial ITS profiling download page of our website (http://probiogenomics.unipr.it/pbi/). Briefly, 89.7% of the aligned 16S rRNA sequences and 85.0% of aligned 23S rRNA sequences reported a perfect match, while an additional 8.2% and 11.8% showed a single mismatch with the 16S rRNA and 23S rRNA genes. Remarkably, just 1.1% of 16S rRNA and 0.3% of 23S rRNA sequences showed ≥3 mismatches, ensuring broad amplification coverage of all known bacterial taxa (Table S1). Moreover, an additional validation step was applied through the PrimerProspector (14) analyze_primers.py and taxa_coverage.py application using default settings and the SILVA NR99 database v.132 (15) in FASTA format. Only 16S rRNA gene sequences of >1,500 bp were used, in order to ensure coverage of the 3′ end. Notably, results revealed average taxonomic coverage of 95.64% and 94.92% for UNI_ITS_fw alignment to16S sequences and UNI_ITS_rv alignment to 23S sequences, respectively, and an absence of rank-specific biases (Data Set S1).

10.1128/mSystems.00404-20.3 TABLE S1 Percentages of reads mapped to the SILVA SSU and LSU databases. Download Table S1, DOCX file, 0.02 MB. Copyright © 2020 Milani et al. 2020 Milani et al. This content is distributed under the terms of the Creative Commons Attribution 4.0 International license.

In this context, nonmicrobial low-biomass PCR amplification would require a higher number of mismatches, as revealed by PrimerProspector (14) analyze_primers.py for Homo sapiens, Mus musculus, and Arabidopsis thaliana nuclear and mitochondrial DNA as well as Arabidopsis thaliana for chloroplast DNA (Data Set S1).To allow easy integration of the methodology into the QIIME v.2 analysis pipeline, we developed a ready-to-use bash script for Linux and MacOS operating systems along with a preformatted database in artifact format for taxonomic classification in a QIIME 2 environment (http://probiogenomics.unipr.it/pbi/) (20, 21). Notably, all ITS-profiling analyses reported in this study were performed using QIIME 2 v.2018.11, but the package available on our website has also been tested to be compatible with QIIME 2 v. 2020.2 and QIIME 2 v. 2020.6, i.e., the latest release available at this time. A guide to using this script is available in Text S1. Briefly, fastq files are filtered to remove reads without the forward primer UNI_ITS_fw. DADA2 (22) is then employed to denoise sequences, dereplicate sequence variants, and remove chimeras, and the amplicon sequence variants (ASVs) obtained are classified at the species level using the QIIME 2 feature-classifier classify-consensus-vsearch (23) method. Furthermore, alpha and beta diversity analyses using multiple statistical metrics are performed.

10.1128/mSystems.00404-20.1 TEXT S1 User manual for the analysis script available at http://probiogenomics.unipr.it/pbi/, discussion of the comparison between ITS profiling and shotgun metagenomics results, and detailed discussion of ITS profiling data obtained from the clinical test case encompassing colorectal cancer biopsy specimens. Download Text S1, DOCX file, 0.1 MB. Copyright © 2020 Milani et al. 2020 Milani et al. This content is distributed under the terms of the Creative Commons Attribution 4.0 International license.

A group of six data sets in fastq format for testing purposes is also available in the “Test” package that can be downloaded from our website (http://probiogenomics.unipr.it/pbi/). These data sets consist of ITS amplicon data obtained from analysis of the bacterial communities harbored by human saliva, human vagina, and cheese samples and are accompanied by an Excel file reporting the expected taxonomic profiles.

### *In silico* evaluation of profiling performances.

In order to evaluate the efficacy of the proposed analysis pipeline across all taxonomic ranks covered by the reference database, we developed three artificial data sets constituted by UNI_ITS_fw/UNI_ITS_rv 250-bp forward amplicons. Each of these data sets included sequences from 500 different randomly selected species, and they were named 500_A, 500_B, and 500_C (Data Set S2). Analysis of these artificial communities revealed that 97.4% and 87.87% of the amplicons were correctly classified at the genus and species levels, respectively, and confirmed the absence of biases in accuracy of specific taxonomic ranks (Data Set S2).

10.1128/mSystems.00404-20.6 DATA SET S2 Results obtained from the analysis of artificial datasets. Download Data Set S2, XLSX file, 0.3 MB. Copyright © 2020 Milani et al. 2020 Milani et al. This content is distributed under the terms of the Creative Commons Attribution 4.0 International license.

**FIG 2:**
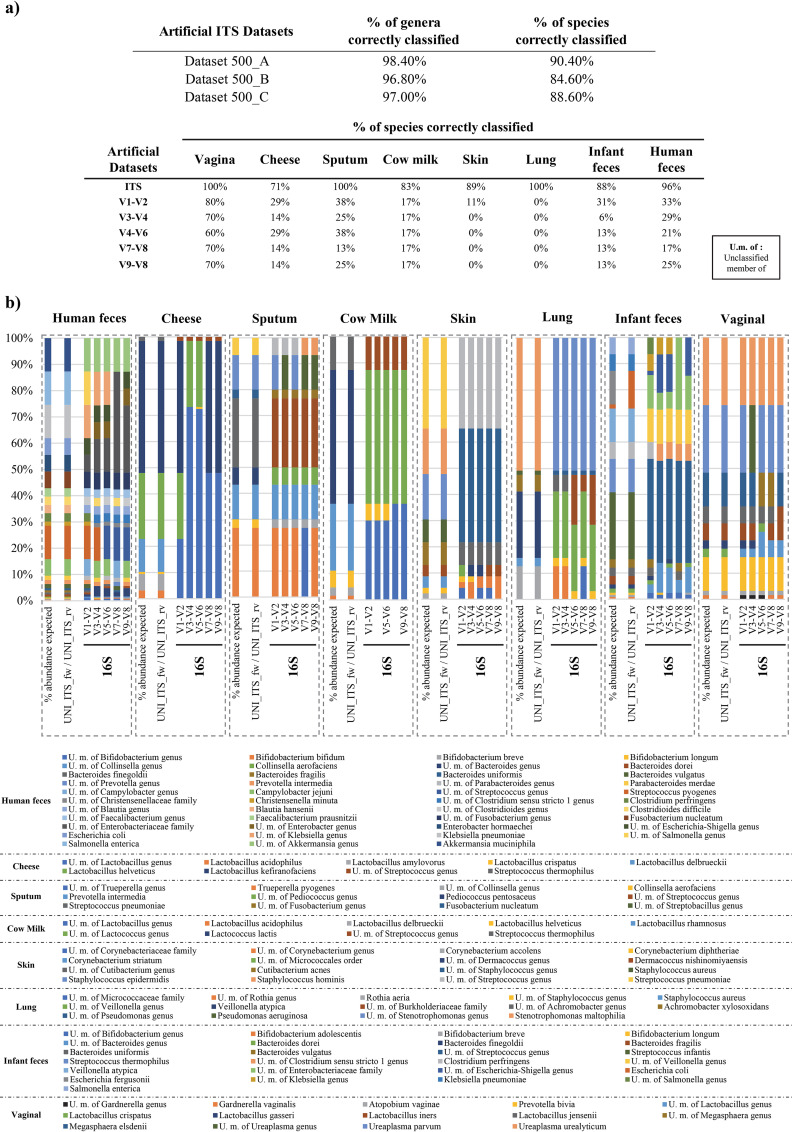
*In silico* evaluation of microbial ITS profiling performance and comparison respect to different hypervariable 16S rRNA gene regions. (a) Percentage of bacterial taxa correctly classified by ITS and 16S rRNA gene profiling methods with respect to expected taxonomic profiles of artificial data sets. (b) Taxonomic profiles observed using ITS and 16S rRNA gene profiling methods and their comparison with respect to expected taxonomic profiles of artificial data sets.

Furthermore, we generated artificial communities constituted by UNI_ITS_fw/UNI_ITS_rv 250 bp forward amplicons obtained from genomes of species representative of bacterial communities typically found in biological matrices of high scientific relevance, i.e., human feces from adults and infants, vagina, sputum, lung, and skin, as well as cow’s milk and cheese. Moreover, the same genomes were used to predict 450-bp amplicons, representing joined 250-bp paired-end reads with 50-bp overlaps, corresponding to multiple 16S rRNA gene variable regions. In detail, 450 bp starting from primers 27F (3′-AGAGTTTGATCMTGGCTCAG-5′), ProbioUni_fw (3′-CCTACGGGRSGCAGCAG-5′), 785F (3′-GGATTAGATACCCTGGTA-5′), 1100F (3′-YAACGAGCGCAACCC-5′), and 1492R (3′-CGGTTACCTTGTTACGACTT-5′) were used to generate artificial data sets covering the V1-V2, V3-V4, V4-V6, V7-V8, and V9-V8 regions of the 16S rRNA gene. The list of species used for each matrix was derived from recent literature (5, 24–35) and shotgun metagenomic analyses reported in this study (see below). Amplicons corresponding to each species were added to each data set at specific relative abundances, reported as expected profiles in Data Set S2, for a total of approximately 50,000 reads. 16S rRNA-based data sets were processed using the 16S rRNA database SILVA v.132 and the sklearn classifier (36) with default settings in a Qiime2 v. 2020.02 environment (20, 21). Remarkably, the results obtained revealed that the profiles generated by ITS profiling match the expected taxonomic profiles, with a species-level classification accuracy of 93.5% (Data Set S2; Fig. 2). In contrast, 16S rRNA gene profiling showed lower accuracy of species-level taxonomic reconstruction. In detail, V1-V2, V3-V4, V4-V6, V7-V8, and V9-V8 regions of the 16S rRNA gene allowed a classification of 30%, 20%, 22%, 18%, and 20%, respectively, of the species across the analyzed artificial data sets (Data Set S2; Fig. 2). While these data clearly demonstrate the higher taxonomic resolution of ITS profiling approach with respect to 16S rRNA gene microbial profiling, it is worth mentioning that synthetic data sets generated and used for test purposes in this study encompass the same sequences present in the databases, since both were retrieved from publicly available genomes. Thus, the reported performances may vary in real settings and the evaluation of the impact of sequence variability with respect to database might require additional *ad hoc* testing.

### Specificity assessment through profiling of an artificial community.

To assess specificity, we performed microbial ITS and 16S rRNA gene profiling of an artificial bacterial community composed of 26 species in equal viable-cell numbers, including multiple species of the same genus (Table S2; Data Set S3). 16S rRNA gene-based profiling was performed using a primer set with broad taxonomic coverage, as shown by comparison with other widely used primers (29, 37–39). Remarkably, microbial ITS profiling successfully identified all species within the artificial community (Data Set S3), whereas 16S rRNA gene profiling was unable to classify at the species level or misclassified 16 of the 26 (61.5%) bacterial species (Data Set S3). While a number of misclassifications observed in the 16S rRNA profiling analysis may be due to issues in the SILVA database v.132, overall, these data confirm higher precision and taxonomic resolution of the ITS-based approach versus those achieved by 16S rRNA-based cataloguing.

10.1128/mSystems.00404-20.4 TABLE S2 Quality filtering table of samples analyzed in this study. Download Table S2, DOCX file, 0.03 MB. Copyright © 2020 Milani et al. 2020 Milani et al. This content is distributed under the terms of the Creative Commons Attribution 4.0 International license.

10.1128/mSystems.00404-20.7 DATA SET S3 Results obtained from analysis of the artificial community. Download Data Set S3, XLSX file, 0.01 MB. Copyright © 2020 Milani et al. 2020 Milani et al. This content is distributed under the terms of the Creative Commons Attribution 4.0 International license.

### Evaluation of profiling performance with biological samples.

**FIG 3:**
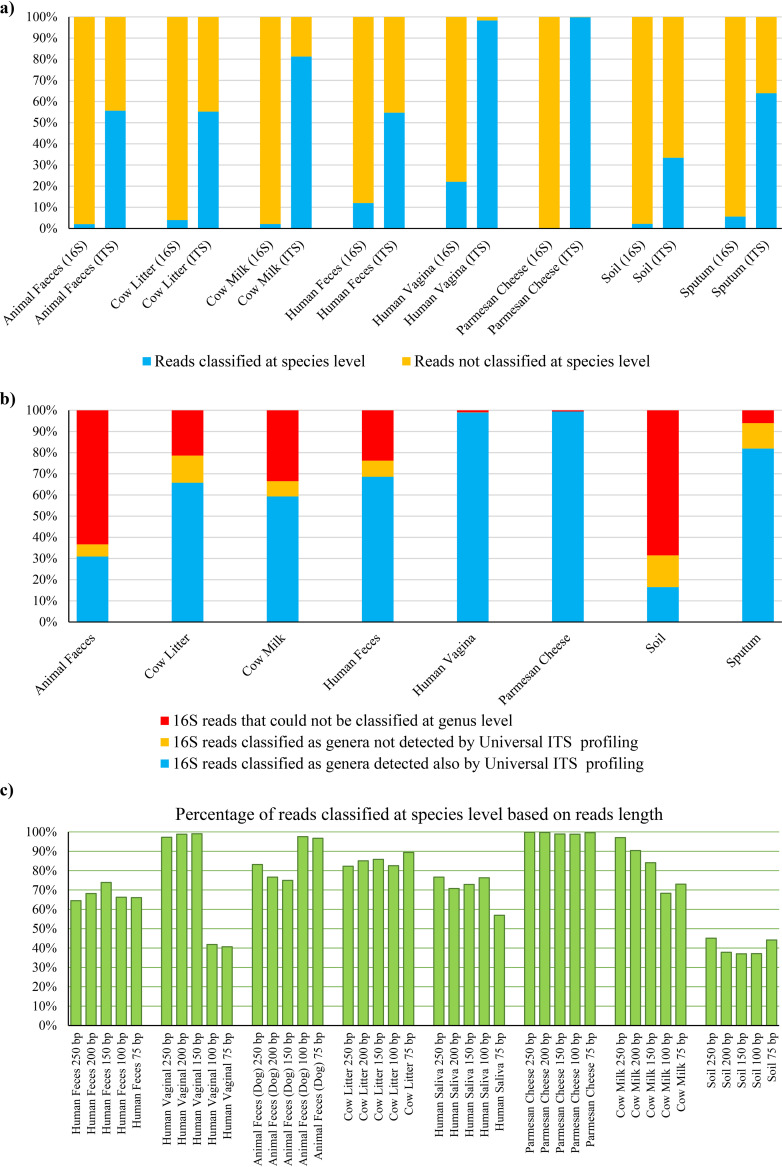
Performance of microbial ITS profiling compared to 16S rRNA gene cataloguing and read-based classification of shotgun metagenomic data sets. (a) Average percentage of reads classified to the species level using ITS and 16S rRNA gene profiling for each matrix analyzed. Data obtained for each sample are reported in Data Set S4. (b) Average percentage of reads classified by 16S rRNA gene profiling as a genus also detected by microbial ITS profiling of the same read, for each matrix analyzed. Data obtained for each sample are reported in Data Set S4. (c) Percentage of reads classified at the species level based on read length.

Performance of the microbial ITS profiling pipeline was furthermore tested through analysis of five samples that cover eight different matrices representing both environmental and host-associated microbial populations (Table S2). Alpha diversity analysis through rarefaction curves built up to 10,000 reads revealed that all matrices reach a plateau (Data Set S4), indicating that the microbial ITS profiling approach requires sequencing depths and costs that are comparable with those of 16S rRNA gene profiling. Remarkably, an overall average of 67.8% of the reads, and up to 100%, were successfully classified at the species level (Fig. 3; Data Set S4). Of note, the discriminatory power of microbial ITS-based profiling is dependent on the availability of reference ITSs extracted from genomic sequences, similar to profiling methods based on shotgun metagenomics data (7, 8, 40–42). Therefore, the percentage of classified species is expected to increase due to ongoing genome decoding of novel bacterial species.

10.1128/mSystems.00404-20.8 DATA SET S4 Results obtained from the analysis of real biological samples. Download Data Set S4, XLSX file, 1.3 MB. Copyright © 2020 Milani et al. 2020 Milani et al. This content is distributed under the terms of the Creative Commons Attribution 4.0 International license.

Microbial compositional data obtained with microbial ITS profiling were compared to those from 16S rRNA gene microbial profiling using *de novo* ASV generation (at 100% identity) and classification with the feature-classifier classify-sklearn method (43) and the SILVA database v. 132, as suggested by the QIIME v.2 manual (44) (Table S2). Notably, comparison of ITS and 16S rRNA gene-based profiles at the genus level revealed that the two approaches have comparable performances at this taxonomic rank: an average of 84.0% of the reads that were classified at the genus level by 16S rRNA gene profiling were assigned to the same taxonomic rank by the microbial ITS approach, while an average of 85.9% of reads classified at the genus level by microbial ITS profiling showed a taxonomy also reported by 16S rRNA profiling (Fig. 3; Data Set S4). Conversely, the average percentage of reads classified at the species level by 16S rRNA gene profiling is 6.2%, which is substantially lower than that obtained by ITS profiling (67.8%) (Fig. 3; Data Set S4). In this context, one should keep in mind that the limited number of taxa properly profiled by 16S rRNA sequences, yet not detected by ITS, is mostly due to the current wider taxonomic coverage of 16S rRNA data sets than of the ITS database, which was generated from available microbial genomes with a validated ribosomal locus. Nevertheless, as argued above, the number of species classified by the ITS profiling approach is expected to increase due to ongoing genome decoding of novel bacterial species. A comparison of microbial ITS profiling with taxonomic reconstruction from shotgun metagenomics data is available in Text S1.

Furthermore, data sets consisting of 250-bp reads obtained from ITS amplicons of a given sample and representing each of the eight profiled biological matrices were shortened to generate artificial ITS amplicon data sets with read lengths of 200, 150, 100, or 75 bp in order to determine the minimum read length needed to achieve sufficient coverage of the ITS region for optimal taxonomic classification. Intriguingly, the data collected indicated that reads with a length of ≥150 bp provided comparable resolution and discriminatory power, as indicated by average percentages of reads classified at the species level of 80.7%, 78.4%, and 78.3% for data at 250 bp, 200 bp, and 150 bp, respectively (Data Set S4; Fig. 3). In contrast, 100-bp and 75-bp reads showed a percentage of reads classified at the species level lower than 71.1% (Data Set S4; Fig. 3).

The microbial ITS profiling pipeline was employed to assess colon biopsy specimens to identify microbial biomarkers of colorectal cancer (CRC). In detail, two mucosal samples were collected from the same region of the colon from 15 individuals diagnosed with CRC, corresponding to adenocarcinoma and healthy mucosa (Data Set S4; Fig. S1). Analysis of microbial ITS data at the species level revealed that Fusobacterium nucleatum, which has been proposed as a key microbial marker of CRC (45, 46), is present at higher relative abundance in adenocarcinoma mucosa in all cases (Fig. S1). Moreover, Fusobacterium hwasookii, Fusobacterium periodonticum, Clostridium chauvoei, and Lactobacillus oris were also observed to be present at higher relative abundances in adenocarcinoma than in healthy mucosa in all individuals in which these species were detected; thus, they may represent additional microbial biomarkers of CRC (Fig. S1). Though performing colonoscopies for biopsy sample collection obviates the need for profiling resident microbiota for CRC detection, the identification of above-mentioned biomarkers at the species level is important for the establishment of a CRC diagnostic protocol based on fecal microbiota analysis. In this context, profiling of the fecal microbiota followed by quantitative normalization by total bacterial cell counts obtained from qPCR or cytofluorimetric assays (47) or the use of novel approaches such as biosensors for rapid quantitative detection of biomarker-specific DNA in feces may represent key tools for cost-effective population screenings.

10.1128/mSystems.00404-20.2 FIG S1 Identification of microbial biomarkers of colorectal cancer based on species-level data. The graphical representation reports the bacterial species that, when detected in the mucosal samples analyzed, showed higher abundance in adenocarcinoma than in healthy mucosa of the same individual. Download FIG S1, PDF file, 0.2 MB. Copyright © 2020 Milani et al. 2020 Milani et al. This content is distributed under the terms of the Creative Commons Attribution 4.0 International license.

### Conclusions.

The currently available methods for complex bacterial community profiling do not allow a cost-effective reconstruction of the bacterial population at the species level. For this reason, we developed a comprehensive ITS-based pipeline for profiling at the (sub)species level that encompasses a bacterial ITS database, optimized universal primers for bacteria, and a ready-to-use bioinformatic script. Taxonomic profiling performance was tested through analysis of an artificial community as well as five different host-associated, food, or environmental matrices, and the data obtained were compared with genus-level profiles obtained by means of 16S rRNA gene microbial profiling. Moreover, the efficacy of this novel approach was also validated in a clinical test case consisting of screening of 15 individuals affected by CRC for bacterial species representing known microbial biomarkers. Intriguingly, accurate species-level data also led to the identification of novel biomarkers of CRC.

Based on collected results, the microbial ITS profiling approach represents an innovative tool for cost-effective and accurate identification and screening of microbial biomarkers at the (sub)species level with relevant impact in research, industrial, and clinical settings.

## MATERIALS AND METHODS

### Ethics approval and consent to participate.

All experimental procedures and protocols involving animals were approved by the Veterinarian Animal Care and Use Committee of Parma University and conducted in accordance with the European Community Council Directives dated 22 September 2010 (2010/63/UE). Human participants gave informed written consent before enrollment. Analyses of human samples were performed in the framework of previous studies approved by the Comitato Etico per Parma. All investigations were carried out following the principles of the Declaration of Helsinki.

### Sample collection.

For the purpose of this study, a total of 70 samples were collected, encompassing 30 colonoscopic biopsy specimens and five biological samples of each of the following matrices: human and animal feces, human vaginal swabs, human sputum, soil, litter from dairy cattle’s husbandries, Parmesan cheese, and dairy cattle milk (Table S2). In detail, by means of endoscopy biopsy forceps, two biopsy samples, one from the adenocarcinoma area and one from the adjacent healthy mucosa, were collected from 15 patients affected by colorectal cancer. Human and animal feces were collected immediately after defecation. Humans and animals included in this study had not taken antibiotics during the previous 6 months. Cow milk samples were harvested directly by hand during milking, after the cow teat ends were cleaned and disinfected, while Parmesan cheese samples were retrieved by trimming fresh Parmesan cheeses. Furthermore, litters were recovered from the ground of different husbandries, while soil samples were collected from different fields. In all cases, immediately after collection, samples were kept on ice and shipped frozen to the laboratory, where they were preserved at −80°C, until they were processed.

Human and animal fecal samples together with sputum and litter samples were subjected to DNA extraction using the QIAamp DNA stool minikit (Qiagen, Germany). A ZymoBIOMICS DNA miniprep kit (Zymo Research Corporation, USA) was used for DNA extraction from vaginal swabs, while DNA extraction from milk and Parmesan cheese samples was performed using the DNeasy mastitis minikit (Qiagen, Germany). DNA from colonoscopic biopsy specimens were extracted using the AllPrep PowerViral DNA/RNA kit (Qiagen, Germany), while the DNeasy PowerSoil kit (Qiagen, Germany) was used for DNA extraction from soil samples. In all cases, the DNA extractions were performed following the manufacturers’ instructions.

### 16S rRNA gene sequencing.

Partial 16S rRNA gene sequences were amplified from extracted DNA using the primer pair Probio_Uni (5′-CCTACGGGRSGCAGCAG-3′) and Probio_Rev (5′-ATTACCGCGGCTGCT-3′) targeting the V3 region of the 16S rRNA gene sequence (37). Illumina adapter overhang nucleotide sequences were added to the partial 16S rRNA gene-specific amplicons, which were further processed by means of the 16S metagenomic sequencing library preparation protocol (part 15044223, rev. B; Illumina). Amplifications were carried out using a Verity thermocycler (Applied Biosystems). The integrity of the PCR amplicons was analyzed by electrophoresis on a 2200 TapeStation instrument (Agilent Technologies, USA). DNA products obtained following PCR-mediated amplification of the 16S rRNA gene sequences were purified by a magnetic purification step employing Agencourt AMPure XP DNA purification beads (Beckman Coulter Genomics GmbH, Bernried, Germany) in order to remove primer dimers. DNA concentration of the amplified sequence library was determined by a fluorometric Qubit quantification system (Life Technologies, USA). Amplicons were diluted to a concentration of 4 nM, and 5-μl quantities of each diluted DNA amplicon sample were mixed to prepare the pooled final library. Sequencing was performed using an Illumina MiSeq sequencer with MiSeq reagent kit v3 chemicals.

### Microbial ITS profiling.

ITS sequences were amplified from extracted DNA using the primer pair UNI_ITS_fw (5′-KRGGRYKAAGTCGTAACAAG-3′) and UNI_ITS_rv (5′-TTTTCRYCTTTCCCTCACGG-3′), targeting the entire spacer region between the 16S rRNA and 23 rRNA genes within the rRNA locus. The amplification was carried out using GoTaq G2 Hot Start polymerase (Promega, USA) on a Verity thermocycler (Applied Biosystems, USA) according to the following protocol: 95°C for 10 min, followed by 32 cycles of 95°C for 1 min, 52°C for 1 min, and 72°C for 1 min and a final step of 72°C for 5 min. The integrity of PCR amplicons was analyzed by gel electrophoresis. The library of ITS amplicons was prepared according to the 16S metagenomic sequencing library preparation protocol (part 15044223, rev. B; Illumina) with modifications in the purification steps. Specifically, the first purification involved 15 μl of Agencourt AMPure XP DNA purification beads (Beckman Coulter Genomics GmbH, Bernried, Germany) in order to remove primer dimers. Then, the second purification step was performed using 30 μl of the above-mentioned purification beads. Sequencing was performed using an Illumina MiSeq sequencer with MiSeq reagent kit v3 chemicals, using 300 cycles.

### 16S rRNA microbial profiling analysis.

The fastq files were processed using a custom script based on the QIIME2 software suite (20, 44). Paired-end read pairs were assembled to reconstruct the complete Probio_Uni/Probio_Rev amplicons. Quality control retained sequences with a length between 140 and 400 bp and mean sequence quality score of >20, while sequences with homopolymers of >7 bp and mismatched primers were removed to reduce quality issues (though Illumina sequencing is not known to be affected by homopolymer sequences). In order to calculate downstream diversity measures, 16S rRNA amplicon sequence variants (ASVs) were defined at 100% sequence homology using DADA2 (22); ASVs not encompassing at least two sequences of the same sample were removed. Notably, this approach allows highly distinctive taxonomic classification at single nucleotide accuracy (20). All reads were classified to the lowest possible taxonomic rank using QIIME2 (20, 44) and a reference data set from the SILVA database (v. 132) (15). Biodiversity within a given sample (alpha diversity) was calculated considering the observed ASVs for 10 subsamplings of the total read pool.

### Microbial ITS profiling analysis.

The fastq files were processed using a custom script based on the QIIME software suite (20, 44). The forward read obtained from paired-end sequencing, corresponding to the UNI_ITS_fw amplicon, was used for taxonomic reconstruction. Quality control retained sequences with a quality score of >20, while sequences with homopolymers of >7 bp and mismatched UNI_ITS_fw primer were omitted. In order to calculate downstream diversity measures, ITS ASVs were defined at 100% sequence homology using DADA2 (22); ASVs not encompassing at least two sequences of the same sample were removed. Notably, this approach allows highly distinctive taxonomic classification at single-nucleotide accuracy (20). All reads were classified to the lowest possible taxonomic rank using QIIME2 (20, 44) and the reference data set UMID (probiogenomics.unipr.it/pbi). Biodiversity within a given sample (alpha diversity) was calculated considering the observed ASVs for 10 subsamplings of the total read pool.

### Shotgun metagenomics.

The extracted DNA was prepared following the Illumina Nextera XT DNA library preparation kit. Briefly, the DNA samples were enzymatically fragmented, barcoded, and purified by using magnetic beads. Samples were then quantified using fluorometric Qubit quantification system (Life Technologies, USA), loaded on a 2200 Tape Station instrument (Agilent Technologies, USA), and normalized to 4 nM. Sequencing was performed using an Illumina NextSeq 500 sequencer with NextSeq High Output v2 kit chemicals for 150 cycles.

### Analysis of metagenomic data sets.

The obtained fastq files were filtered for reads with a quality of <25, for reads of >80 bp. Moreover, bases were removed from the end of the reads unless the average quality score was >25, in a window of 5 bp. Quality-filtered data were used to further analysis with METAnnotatorX (8) and MetaPhlAn2 (42) for taxonomic profile reconstruction. Both software packages were used with default settings, and the RefSeq database obtained from NCBI in October 2019 was used for taxonomic classification using METAnnotatorX (8).

### Data availability.

Raw sequences of Universal ITS and 16S rRNA gene profiling data coupled with shotgun metagenomics data are accessible through BioProject accession number PRJNA562817.

10.1128/mSystems.00404-20.9 DATA SET S5 Alignment file in Clustal format showing alignment of 100 16S rRNA gene sequences used to identify the optimal conserved region for primer design. Download Data Set S5, TXT file, 0.8 MB. Copyright © 2020 Milani et al. 2020 Milani et al. This content is distributed under the terms of the Creative Commons Attribution 4.0 International license.

10.1128/mSystems.00404-20.10 DATA SET S6 Alignment file in Clustal format showing alignment of 100 23S rRNA gene sequences used to identify the optimal conserved region for primer design. Download Data Set S6, TXT file, 1.1 MB. Copyright © 2020 Milani et al. 2020 Milani et al. This content is distributed under the terms of the Creative Commons Attribution 4.0 International license.
