# Supplementary material for: Untangling Species-Level Composition of Complex Bacterial Communities through a Novel Metagenomic Approach
Source: mSystems. 2020 Jul 28;5(4):e00404-20. doi: 10.1128/mSystems.00404-20 (PMC7394355; doi:10.1128/mSystems.00404-20)
Supplement: TABLE S1 [file mSystems.00404-20-st001.docx]

| **Table S1: Percentages of reads mapped to the SILVA SSU and LSU databases.** | | |
| --- | --- | --- |
|  | **Database used for alignment** | |
| **n° of missmatches** | **SILVA SSU (16S) NR v.132** | **SILVA LSU (23S) NR v.132** |
| 0 | 89.71% | 84.99% |
| 1 | 8.17% | 11.77% |
| 2 | 0.98% | 2.91% |
| 3 | 0.53% | 0.20% |
| 4 | 0.45% | 0.09% |
| 5 | 0.15% | 0.04% |
