# Supplementary material for: Untangling Species-Level Composition of Complex Bacterial Communities through a Novel Metagenomic Approach
Source: mSystems. 2020 Jul 28;5(4):e00404-20. doi: 10.1128/mSystems.00404-20 (PMC7394355; doi:10.1128/mSystems.00404-20)
Supplement: TEXT S1 [file mSystems.00404-20-s0001.docx]

**Untangling species-level composition of complex bacterial communities through a novel metagenomic approach**

Key words: metagenomics, ITS, profiling

Christian Milani^1,2^, Giulia Alessandri^3^, Marta Mangifesta^1^, Leonardo Mancabelli^1^, Gabriele Andrea Lugli^1^, Federico Fontana^1^, Giulia Longhi^1,4^, Rosaria Anzalone^4^, Alice Viappiani^4^, Sabrina Duranti^1^, Francesca Turroni^1,2^, Renato Costi^5^, Alfredo Annicchiarico^5^, Andrea Morini^5^, Leopoldo Sarli^5^, Maria Cristina Ossiprandi^2,3^, Douwe van Sinderen^6^ and Marco Ventura^1,2^

Laboratory of Probiogenomics, Department of Chemistry, Life Sciences, and Environmental Sustainability, University of Parma, Parma, Italy^1^; Microbiome Research Hub, University of Parma, Parma, Italy^2^; Department of Veterinary Medical Science, University of Parma, Parma, Italy^3^; GenProbio srl, Parma, Italy^4^; Department of Medicine and Surgery, University of Parma, Parma, Italy^5^; APC Microbiome Institute and School of Microbiology, Bioscience Institute, National University of Ireland, Cork, Ireland^6^

Correspondence. Mailing address for Marco Ventura Laboratory of Probiogenomics, Department of Chemistry, Life Sciences, and Environmental Sustainability, University of Parma, Parco Area delle Scienze 11a, 43124 Parma, Italy. Phone: ++39-521-905666. Fax: ++39-521-905604. E-mail: [marco.ventura@unipr.it](mailto:marco.ventura@unipr.it)

**User Manual**

Installation

The Microbial ITS profiling analysis package can be downloaded from the website “http://probiogenomics.unipr.it/pbi/” as a .zip file containing:

- Bacterial_ITS_annotator.sh: the bash script that performs the Microbial ITS profiling analysis.
- “database” folder: folder containing the Microbial ITS database.
- “fastq_input” folder: folder where input fastq files needs to be placed.

These three elements must be placed in the same folder in a linux environment with Qiime2 installed. All the dependencies/software needed to run the Bacterial_ITS_annotator.sh bash script are included in Qiime2.

Usage

In order to perform the Bacterial ITS profiling analysis, a single .fastq file for each sample must be placed in the “fastq_input” folder. In case paired-end sequencing was used, the .fastq file provided in the “fastq_input” folder must correspond to reads starting with primer UNI_ITS_fw (5'-KRGGRYKAAGTCGTAACAAG-3') covering the 3’-end of the 16S rRNA sequence.

The script can be executed in the linux console simply by moving to the folder path where the script is located and by typing “./Bacterial_ITS_annotator.sh”.

Console text will notify the user that the “Fasting_Map.txt” and “sample-metadata.tsv” file has been created and allows the user to add to this file additional columns with samples’ metadata that will be used for downstream analyses (e.g. PCoA representation of beta-diversity) before continue the execution of the script.

Console messages will report the ongoing analyses performed by the script.

A log file named “Qiime2_log.txt” is also created.

Results

Upon completion of the pipeline by the Bacterial_ITS_annotator.sh script, the filtered .fastq files will be placed in the “fastq_input_filtered” folder and all temporary files generated along the pipeline are moved in the “data_analysis” folder.

Furthermore, results will be placed in the “results” folder. In detail the “results” folder contains:

- “alpha_diversity” folder: results of the alpha diversity analysis using the Simpson, Shannon, Faith, Chao1 and Observed OTUs indexes.
- “beta_diversity” folder: results of the beta diversity analysis using the unweighted UniFrac, weighted UniFrac and Bray Curtis indexes.
- “taxonomy” folder: results of the taxonomic profiling at Phylum, Family, Genus and Species level.
- “ASVs” folder: results of the ASVs analysis encompassing the reference sequence of each ASVs (ASVs_rep_seqs.fasta), the taxonomy attributed at each ASVs (ASVs_taxonomy.txt) and the table of ASVs abundance for each sample (ASVs_table.txt).
- “forward_primer_screening_stats.txt” file: results of the UNI_ITS_fw primer screening through cutadapt.
- “filtering_table.txt” file: filtering table of all the analysed samples.

Notes

- OTUs can be considered Amplicon Sequence Variants, ASVs, since these are generated at 100% identity by this analysis.
- If no metadata is added to the “sample-metadata.tsv” files, the script will be completed but few analyses will be skipped.
- Alpha-diversity analysis is set to reach a maximum subsampling of 30,000 reads. In case no sample reaches this depth, the analysis will be skipped. The user can lower this value at line 151 of the Bacterial_ITS_annotator.sh script, by changing the option “--p-max-depth 30000”.
- Sensitivity and Specificity of the taxonomic classification can be adjusted by the user by editing command options at line 156 of the Bacterial_ITS_annotator.sh script. Details are reported in the Qiime2 manual (https://docs.qiime2.org) at the “qiime feature-classifier” section.

**Comparison of ITS profiling results with shotgun metagenomics.** A sample for each of the eight texted matrices was submitted to shotgun sequencing followed by reads-based taxonomic profiling based on NCBI nr database [using METAnnotatorX (1)] and the MetaPhlAn2 database [using MetaPhlAn2 (2)] (Table S2) (Supplementary Excel file 3). Comparison of shotgun-based and ITS-based results revealed that species profiled using METAnnotatorX and MetaPhlAn2 cover, on average, respectively only the 53.2 % and 32.8 % of the profiles retrieved by Microbial ITS profiling (Supplementary Excel file 3). This is explained by the fact that shotgun-based approaches provide profiles representing the total population of reads that could be attributed to species for which sequenced genomes are available in public databases, while unaligned reads are ignored in the final output. In contrast, methods based on amplification of a specific marker gene, e.g. Microbial ITS profiling, provide profiles normalized with respect to the whole input dataset since the latter is constituted solely by amplicons derived by amplification of the same ubiquitous genomic region. Thus, allowing for reads that could not be classified at species level to be attributed to higher taxonomic ranks through phylogenetic inference, leading to a comprehensive representation of the bacterial population.

**Colorectal cancer clinical test case****.** The Microbial ITS profiling pipeline was employed to assess colon biopsies to identify microbial biomarkers of colorectal cancer (CRC). In detail, two mucosal samples were collected from the same region of the colon of 15 individuals diagnosed with CRC, corresponding to adenocarcinoma and healthy mucosa (Supplementary Excel file 4) (Figure S1). Analysis of Microbial ITS data at species level revealed that 10 individuals harbour *Fusobacterium nucleatum* (Figure S1), which has previously been proposed as a key microbial marker of CRC (3,4). Moreover, when *F. nucleatum* is present in the gut environment, its relative abundance is higher in adenocarcinoma mucosa in all cases (Figure S1). In this context, *Fusobacterium hwasookii*, *Fusobacterium periodonticum*, *Clostridium chauvoei* and *Lactobacillus oris* were also observed to be present at a higher relative abundance in adenocarcinoma when compared to heathy mucosa in all individuals in which these species were detected, which may thus represent additional microbial biomarkers of CRC (Figure S1). Furthermore, Figure 2 shows that *Streptococcus sanguinis* and *Pediococcus pentosaceus* are present at higher abundance in adenocarcinoma mucosa in > 83 % of individuals in which they were detected.

**References**

1 Milani, C. *et al.* Tracing mother-infant transmission of bacteriophages by means of a novel analytical tool for shotgun metagenomic datasets: METAnnotatorX. *Microbiome* **6**, 145, doi:10.1186/s40168-018-0527-z (2018).

2 Truong, D. T. *et al.* MetaPhlAn2 for enhanced metagenomic taxonomic profiling. *Nat Methods* **12**, 902-903, doi:10.1038/nmeth.3589 (2015).

3 Brennan, C. A. & Garrett, W. S. Fusobacterium nucleatum - symbiont, opportunist and oncobacterium. *Nat Rev Microbiol* **17**, 156-166, doi:10.1038/s41579-018-0129-6 (2019).

4 Yu, T. *et al.* Fusobacterium nucleatum Promotes Chemoresistance to Colorectal Cancer by Modulating Autophagy. *Cell* **170**, 548-563 e516, doi:10.1016/j.cell.2017.07.008 (2017).
