# Supplementary material for: Untangling Species-Level Composition of Complex Bacterial Communities through a Novel Metagenomic Approach
Source: mSystems. 2020 Jul 28;5(4):e00404-20. doi: 10.1128/mSystems.00404-20 (PMC7394355; doi:10.1128/mSystems.00404-20)
Supplement: TABLE S2 [file mSystems.00404-20-st002.docx]

| **Table S2: Quality filtering table of samples analyzed in this study.** | | | | | | | | |
| --- | --- | --- | --- | --- | --- | --- | --- | --- |
|  | **ITS** | | | **16S** | | | **Shotgun** | |
| **Sample_Name** | **input** | **filtered** | **non-chimeric** | **input** | **filtered** | **non-chimeric** |  |  |
| Artificial_Sample | 53505 | 39268 | 32932 | 10922 | 9282 | 8343 |  |  |
| Animal_Faeces_1 (Dog) | 278722 | 244841 | 229916 | 77169 | 73247 | 70937 | 10815098 | 10606493 |
| Animal_Faeces_2 (Pig) | 74277 | 58035 | 52613 | 71867 | 68571 | 58702 |  |  |
| Animal_Faeces_3 (Dairy Cattle) | 64230 | 49425 | 48876 | 52151 | 50915 | 44304 |  |  |
| Animal_Faeces_4 (Dairy Cattle) | 50014 | 22376 | 22323 | 61633 | 60520 | 55782 |  |  |
| Animal_Faeces_5 (Dairy Cattle) | 21762 | 14417 | 14194 | 496529 | 441518 | 356968 |  |  |
| Cow_Litter_1 | 74222 | 62455 | 60564 | 61879 | 59045 | 56711 |  |  |
| Cow_Litter_2 | 68594 | 55482 | 53808 | 95520 | 91332 | 85601 |  |  |
| Cow_Litter_3 | 20752 | 10941 | 10773 | 68339 | 66322 | 64840 |  |  |
| Cow_Litter_4 | 124525 | 97855 | 93279 | 69623 | 66591 | 64414 |  |  |
| Cow_Litter_5 | 137502 | 103907 | 102687 | 78038 | 74629 | 67549 | 13703634 | 12952556 |
| Cow_Milk_1 | 123369 | 102857 | 97522 | 76653 | 73966 | 72008 |  |  |
| Cow_Milk_2 | 110253 | 85587 | 82024 | 54576 | 52805 | 47966 |  |  |
| Cow_Milk_3 | 21246 | 12569 | 12569 | 38166 | 36955 | 34737 |  |  |
| Cow_Milk_4 | 107525 | 90058 | 85916 | 71698 | 69367 | 65373 | 19144692 | 17745814 |
| Cow_Milk_5 | 6530 | 3420 | 3385 | 42832 | 41654 | 39938 |  |  |
| Human_Feces_1 | 94745 | 71206 | 68444 | 163889 | 157835 | 136001 | 6346296 | 6220201 |
| Human_Feces_2 | 27292 | 16969 | 16454 | 156579 | 139978 | 118953 |  |  |
| Human_Feces_3 | 25240 | 13735 | 12959 | 119114 | 107410 | 90931 |  |  |
| Human_Feces_4 | 17449 | 8808 | 8749 | 66737 | 58259 | 50320 |  |  |
| Human_Feces_5 | 8092 | 5010 | 4835 | 108882 | 97521 | 83399 |  |  |
| Human_Vagina_1 | 18114 | 12109 | 12109 | 30855 | 25871 | 25766 |  |  |
| Human_Vagina_2 | 30456 | 22187 | 20797 | 38125 | 32972 | 27761 |  |  |
| Human_Vagina_3 | 14475 | 9418 | 9292 | 49988 | 43573 | 36393 | 1178927 | 1075689 |
| Human_Vagina_4 | 19687 | 13252 | 13252 | 37096 | 32060 | 30194 |  |  |
| Human_Vagina_5 | 15407 | 10993 | 10594 | 177148 | 155800 | 136883 |  |  |
| Parmesan_Cheese_1 | 65011 | 55166 | 54723 | 69786 | 67260 | 58196 |  |  |
| Parmesan_Cheese_2 | 97904 | 86000 | 84460 | 70230 | 67421 | 57974 |  |  |
| Parmesan_Cheese_3 | 57953 | 48832 | 48537 | 52279 | 50705 | 37689 |  |  |
| Parmesan_Cheese_4 | 97077 | 83297 | 76733 | 85756 | 82290 | 71687 | 25088478 | 22549792 |
| Parmesan_Cheese_5 | 93161 | 73130 | 67123 | 70042 | 67722 | 57436 |  |  |
| Soil_1 | 58719 | 38162 | 37260 | 123378 | 109453 | 105187 | 1113976 | 972942 |
| Soil_2 | 84976 | 66382 | 58162 | 9347 | 8814 | 8139 |  |  |
| Soil_3 | 34890 | 23835 | 17757 | 6643 | 6331 | 6304 |  |  |
| Soil_4 | 52837 | 32171 | 32171 | 43462 | 41207 | 39299 |  |  |
| Soil_5 | 40202 | 24399 | 24399 | 8690 | 8210 | 7948 |  |  |
| Sputum_1 | 16299 | 10340 | 10340 | 54431 | 46842 | 43044 |  |  |
| Sputum_2 | 52212 | 42945 | 40500 | 67340 | 59359 | 55041 |  |  |
| Sputum_3 | 22676 | 15471 | 15250 | 57909 | 49972 | 44952 | 814587 | 760912 |
| Sputum_4 | 21270 | 13763 | 13640 | 5109 | 4367 | 4245 |  |  |
| Sputum_5 | 27340 | 16818 | 16497 | 43611 | 37214 | 34259 |  |  |
| Biopsy_1H | 72843 | 51079 | 49430 |  |  |  |  |  |
| Biopsy_1C | 61898 | 34872 | 34100 |  |  |  |  |  |
| Biopsy_2H | 79871 | 55450 | 54343 |  |  |  |  |  |
| Biopsy_2C | 80848 | 55582 | 55563 |  |  |  |  |  |
| Biopsy_3H | 79541 | 52980 | 52830 |  |  |  |  |  |
| Biopsy_3C | 95026 | 68110 | 67423 |  |  |  |  |  |
| Biopsy_4H | 67823 | 39628 | 38635 |  |  |  |  |  |
| Biopsy_4C | 91004 | 56272 | 55186 |  |  |  |  |  |
| Biopsy_5H | 71088 | 46489 | 44456 |  |  |  |  |  |
| Biopsy_5C | 91505 | 66938 | 66251 |  |  |  |  |  |
| Biopsy_6H | 66331 | 37591 | 36875 |  |  |  |  |  |
| Biopsy_6C | 66890 | 41335 | 40625 |  |  |  |  |  |
| Biopsy_7H | 70873 | 42079 | 41950 |  |  |  |  |  |
| Biopsy_7C | 76757 | 47645 | 47392 |  |  |  |  |  |
| Biopsy_8H | 91611 | 70784 | 70784 |  |  |  |  |  |
| Biopsy_8C | 87124 | 66626 | 66551 |  |  |  |  |  |
| Biopsy_9H | 54636 | 35800 | 35712 |  |  |  |  |  |
| Biopsy_9C | 92080 | 65971 | 63855 |  |  |  |  |  |
| Biopsy_10H | 55012 | 35245 | 35162 |  |  |  |  |  |
| Biopsy_10C | 53194 | 31874 | 31400 |  |  |  |  |  |
| Biopsy_11H | 80145 | 62142 | 61262 |  |  |  |  |  |
| Biopsy_11C | 62033 | 40480 | 39648 |  |  |  |  |  |
| Biopsy_12H | 78014 | 55245 | 54255 |  |  |  |  |  |
| Biopsy_12C | 91859 | 70918 | 69202 |  |  |  |  |  |
| Biopsy_13H | 57825 | 42633 | 42611 |  |  |  |  |  |
| Biopsy_13C | 67904 | 48083 | 48038 |  |  |  |  |  |
| Biopsy_14H | 45325 | 13435 | 13404 |  |  |  |  |  |
| Biopsy_14C | 58088 | 37772 | 35484 |  |  |  |  |  |
| Biopsy_15H | 67935 | 46142 | 45782 |  |  |  |  |  |
| Biopsy_15C | 68661 | 43077 | 42267 |  |  |  |  |  |
