# Supplementary material for: Untangling Species-Level Composition of Complex Bacterial Communities through a Novel Metagenomic Approach
Source: mSystems. 2020 Jul 28;5(4):e00404-20. doi: 10.1128/mSystems.00404-20 (PMC7394355; doi:10.1128/mSystems.00404-20)
Supplement: FIG S1 [file mSystems.00404-20-sf001.pdf]

	Biopsy_1H	Biopsy_1C	Biopsy_2H	Biopsy_2C	Biopsy_3H	Biopsy_3C	Biopsy_4H	Biopsy_4C	Biopsy_5H	Biopsy_5C	Biopsy_6H	Biopsy_6C	Biopsy_7H	Biopsy_7C	Biopsy_8H	Biopsy_8C
Fusobacterium hwasookii	0.00%	2.80%	0.00%	0.00%	0.00%	0.00%	0.56%	0.63%	0.00%	0.00%	0.00%	0.00%	0.00%	0.00%	0.00%	0.00%
Fusobacterium nucleatum	0.59%	8.49%	0.00%	0.00%	0.00%	6.12%	0.32%	2.30%	0.00%	0.23%	0.00%	0.00%	0.41%	3.81%	0.00%	0.00%
Fusobacterium periodonticum	0.00%	0.08%	0.00%	0.00%	0.04%	0.11%	0.00%	0.00%	0.00%	0.01%	0.00%	0.00%	0.04%	0.75%	0.00%	0.00%
Fusobacterium varium	0.00%	0.00%	0.00%	0.00%	0.00%	0.00%	0.00%	0.00%	0.00%	0.00%	0.00%	0.00%	0.00%	0.00%	0.00%	0.00%
Clostridium chauvoei	0.00%	0.00%	0.00%	0.00%	0.00%	0.00%	0.00%	0.00%	0.05%	0.07%	0.01%	0.02%	0.06%	0.22%	0.00%	0.00%
Lactobacillus oris	0.00%	0.00%	0.01%	0.10%	0.00%	0.00%	0.00%	0.00%	0.05%	0.06%	0.00%	0.00%	0.00%	0.00%	0.00%	0.00%
Streptococcus sanguinis	0.15%	0.16%	0.00%	0.00%	1.78%	6.60%	0.00%	0.01%	0.00%	0.03%	0.00%	0.00%	0.00%	0.00%	0.03%	0.45%
Pediococcus pentosaceus	0.03%	0.00%	0.00%	0.00%	0.00%	0.00%	0.00%	0.06%	0.00%	0.13%	0.00%	0.03%	0.36%	0.77%	0.00%	0.00%

	Biopsy_9H	Biopsy_9C	Biopsy_10H	Biopsy_10C	Biopsy_11H	Biopsy_11C	Biopsy_12H	Biopsy_12C	Biopsy_13H	Biopsy_13C	Biopsy_14H	Biopsy_14C	Biopsy_15H	Biopsy_15C
Fusobacterium hwasookii	0.00%	0.00%	0.00%	0.00%	0.00%	0.00%	0.00%	0.00%	0.00%	0.00%	0.00%	0.00%	0.00%	0.00%
Fusobacterium nucleatum	0.05%	15.3%	0.00%	0.00%	0.76%	6.82%	0.00%	0.00%	0.02%	0.07%	0.00%	1.58%	0.10%	0.37%
Fusobacterium periodonticum	0.51%	1.64%	0.00%	0.00%	0.00%	0.00%	0.00%	0.00%	0.00%	0.00%	0.00%	0.00%	0.00%	0.00%
Fusobacterium varium	13.2%	8.35%	0.00%	0.00%	0.00%	0.00%	0.00%	0.00%	0.00%	0.00%	0.51%	26.9%	0.00%	0.00%
Clostridium chauvoei	0.00%	0.00%	0.00%	0.00%	0.00%	0.00%	0.00%	0.00%	0.00%	0.00%	0.24%	1.92%	0.00%	0.04%
Lactobacillus oris	0.00%	0.00%	0.00%	0.00%	0.00%	0.00%	0.00%	0.02%	0.00%	0.00%	0.00%	0.00%	0.00%	0.03%
Streptococcus sanguinis	0.00%	0.05%	0.00%	0.00%	0.00%	0.03%	0.00%	0.00%	0.14%	0.05%	0.00%	0.00%	0.21%	0.42%
Pediococcus pentosaceus	0.00%	0.00%	0.00%	0.00%	0.00%	0.00%	0.04%	0.07%	0.00%	0.00%	0.00%	0.00%	0.00%	0.00%

Higher in adenocarcinoma mucosa

Higher in healthy mucosa

Absent in both

Figure S1
